# Biocontrol Activity of New Lactic Acid Bacteria Isolates Against Fusaria and *Fusarium* Mycotoxins

**DOI:** 10.3390/toxins17020068

**Published:** 2025-02-04

**Authors:** S. Vipin Krishnan, P. A. Anaswara, K. Madhavan Nampoothiri, Szilvia Kovács, Cintia Adácsi, Pál Szarvas, Szabina Király, István Pócsi, Tünde Pusztahelyi

**Affiliations:** 1 Microbial Processes and Technology Division (MPTD), CSIR-National Institute for Interdisciplinary Science and Technology (NIIST), Thiruvananthapuram 695019, India; vipinkrishnan05@gmail.com (S.V.K.); anaswaraprakash001@gmail.com (P.A.A.); 2 Academy of Scientific and Innovative Research (AcSIR), Ghaziabad 201002, India; 3 Food and Environmental Toxicology Research Group, Central Laboratory of Agricultural and Food Products, Faculty of Agricultural and Food Sciences and Environmental Management, University of Debrecen, H-4032 Debrecen, Hungary; kovacs.szilvia@agr.unideb.hu (S.K.); adacsi.cintia@agr.unideb.hu (C.A.); 4 Centre for Agricultural Genomics and Biotechnology, Faculty of Agricultural and Food Sciences and Environmental Management Center, University of Debrecen, H-4400 Nyíregyháza, Hungary; szarvas.pal@agr.unideb.hu; 5 Department of Molecular Biotechnology and Microbiology, Institute of Biotechnology, Faculty of Science and Technology, University of Debrecen, H-4032 Debrecen, Hungary; kiraly.szabina.18@gmail.com (S.K.); pocsi.istvan@science.unideb.hu (I.P.)

**Keywords:** lactic acid bacterium, *Fusarium*, antagonism, mycotoxin, corn, wheat, peanut

## Abstract

As significant fungal pathogens of crops, Fusaria species contaminate various food and feed commodities. Some of the *Fusarium* spp. secondary metabolites (e.g., trichothecenes, zearalenone, and fumonisins) are widely known toxic molecules (mycotoxins) with chronic and acute effects on humans and animals. The growing demand for safer, pesticide-free food drives us to increase biological control during crop growing. Recent research suggests that lactic acid bacteria (LABs) as biocontrol are the best choice for extenuating *Fusarium* mycotoxins. Newly isolated LABs were tested as antifungal agents against *Fusarium verticillioides*, *F. graminearum*, and *F. oxysporum*. The characterized and genetically identified LABs belonged to *Limosilactobacillus fermentum* (SD4) and *Lactiplantibacillus plantarum* (FCW4 and CB2) species. All tested LABs and their cell-free culture supernatants showed antagonism on the MRS solid medium. The antifungal activity was also demonstrated on surface-sterilized wheat and peanuts. The germination test of corn kernels proved that the LAB strains SD4 and FCW4 significantly (*p* < 0.05) enhanced root and shoot development in plantlets while simultaneously suppressing the outgrowth of *F. verticillioides*. Small-scale corn silage fermentation revealed the significant effects of SD4 supplementation (decreased zearalenone, lower mold count, and total reduction of deoxynivalenol) within the mixed populations.

## 1. Introduction

Food- and feed-contaminating filamentous fungi are causing high economic loss and health risks for human and animal consumers. Some low-molecular-weight secondary metabolites of filamentous fungi are known as mycotoxins. Mycotoxins are commonly resistant to a broad spectrum of environmental factors and undergo slow degradation [1]. *Fusarium* filamentous fungi are notorious pathogens of various food grains and seeds [1,2]. Among various mycotoxins, deoxynivalenol (DON), fumonisins (e.g., FB1), zearalenone (ZEA), and T2/HT2 toxins cause harmful effects [1,3].

*Fusarium verticillioides* (teleomorph *Gibberella fujikuroi*) is a primary fungal pathogen of grains and nuts. The fungus can be isolated in cereals as an endophyte, usually causing no disease symptoms. Under propitious conditions like high humidity and temperature or plant stress like insect or fungal attacks, *F. verticillioides* can cause seedling blight, stalk, or ear rot in maize. In addition to reducing crop yield, it produces fumonisin (FUM) mycotoxins harmful to humans and animals [1,4]. *Fusarium graminearum* causes *Fusarium* head blight on grains. It produces type-B trichothecenes like deoxynivalenol (DON) or nivalenol during infection. DON is a virulence factor that promotes the spread of the *Fusarium* head blight [5]. Estrogenic zearalenone (ZEA) is also produced by *F. graminearum* [1]. Soil-borne ascomycete *Fusarium oxysporum* produces fumonisins, fusaric acid and some emerging mycotoxins (e.g., enniatins, beauvericin, moniliformin) [1] and can attack a wide range of crops [6].

Different methods are employed to control the fungal contamination, such as pre- and post-harvesting handling. Chemical preservatives, to some extent, are successful in retarding microbial growth and achieving a considerably longer shelf-life. However, the increasing demand for safer products requires manufacturers to find natural alternatives to replace chemically derived ingredients to guarantee complete food safety.

Traditionally, lactic acid bacteria (LAB) have been used as preservatives in food and animal feed, and their food-preserving effect is mainly due to the formation of organic acids and certain chemicals [3,7,8]. Generally, LABs are deemed Generally Recognized As Safe (GRAS) microbes by the Food and Drug Agency (FDA, USA), and some strains possess a Qualified Presumption of Safety (QPS) from the European Food Safety Authority (EFSA, EU). Therefore, LABs are considered a good choice as natural preservatives to control fungal growth and subsequent mycotoxin production.

The antifungal activity of LAB strains against mycotoxigenic filamentous fungi (e.g., *Fusarium* spp., *Penicillium* spp., *Aspergillus* spp.) may result from competitive growth and the production of a varied spectrum of simple metabolites, such as organic acids (lactic, propionic, and acetic acids), ethanol, hydrogen peroxide, diacetyl, phenyl lactic acid, reuterin, cyclic dipeptides, benzoic acid, hydroxylated fatty acids, methylhydantoin, and mevalonolactone, as well as more complex products like proteinaceous compounds and bacteriocin-like substances [7,8,9,10]. In addition, LABs can remove/reduce mycotoxins in contaminated media through chemical/enzymatic degradation, metabolic conversions, or adsorption processes, through which they bind mycotoxins to their cell-wall components, preventing their absorption into food or feed [10,11,12,13].

The study aimed to identify LAB isolates as biocontrol agents suitable for controlling *Fusarium* mold and reducing *Fusarium* mycotoxins in plant materials.

## 2. Results

### 2.1. Characterization of LABs Capable of Fusarium Antagonism

It is well known that LAB strains produce antifungal compounds in culture media. The antifungal activity of isolated bacteria against *Fusarium*, a well-known mycotoxin producer, was evaluated. Subsequently, the selected antagonistic bacteria were identified and characterized by specific PCR and sequencing of the 16S ribosomal RNA gene.

**Figure 1:**
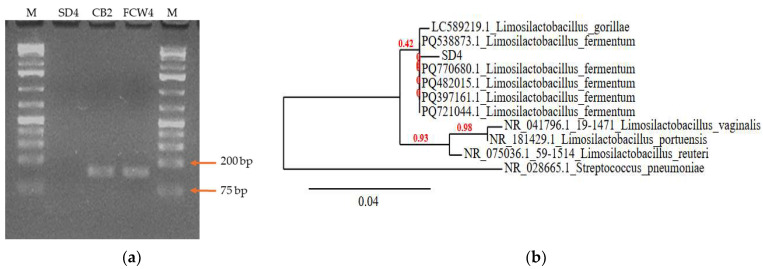
Nucleic acid-based identification of LAB strains. (**a**) Sequence amplification with *Lactiplantibacillus plantarum*-specific primers (Plantarum R-Plantarum F), which are specific for unique LPXTG-motif cell-wall anchor domain protein gene (EFK29584.1), resulted in 147 bp nucleic acid amplificant and revealed FCW4 and CB2 strains as *L. plantarum* isolates. M: 1 kb DNA marker (**b**) Neighbor-joining phylogenetic tree based on 16S rDNA sequence of *Limosilactobacillus fermentum* SD4 showing relationship with other members. *Streptococcus pneumoniae* NR_028665.1 was used as an outgroup. The scale bar indicates the evolutionary distance, and the number along the tree branch indicates the bootstrap value.

The SD4 strain was shown to have 99.69% homology with the *Limosilactobacillus fermentum* BLM1.N strain (PQ770680.1), and FCW4 showed 98.90% identity with *Lactiplantibacillus plantarum* Heal19 (CP055123.1). The strain CB2 showed high homology with *Lactiplantibacillus argentoratensis* (100%) but also with *L. pentosus* (100%) and *L. plantarum* (100%). Therefore, another identification step needed to be introduced. The *L. plantarum*-specific primer pair (Plantarum R -Plantarum F) for the LPXTG-motif cell-wall anchor domain protein gene (EFK29584.1) was applied to confirm species identification. The CB2 and FCW4 strain’s identity was also confirmed with the Plantarum R -Plantarum F specific primer pair (Figure 1a) and was identified as *L. plantarum* (Table 1). For the SD4 strain, the result from the PCR with the 515F-1492R primer pair was verified with the Plantarum R-Plantarum F specific primer pair (Figure 1a). Phylogenetic analysis revealed that the SD4 sequence is closely related to *L. fermentum*-type strains’ sequences (Figure 1b). Sequences of the isolates were submitted to the National Centre for Biotechnology Information (NCBI); the accession numbers are depicted in Table 1.

**Figure 2:**
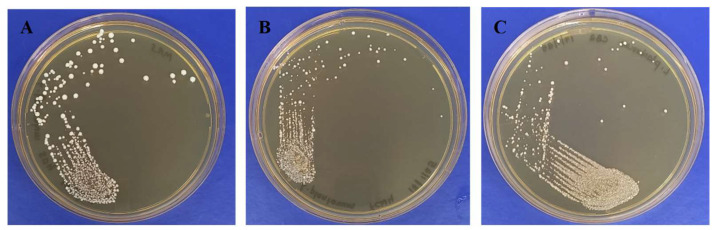
Macromorphology of (**A**) *Limosilactobacillus fermentum* SD4, (**B**) *Lactiplantibacillus plantarum* FCW4, (**C**) *Lactiplantibacillus plantarum* CB2.

LAB identification was also strengthened with biochemical characterization and Gram staining. *L. fermentum* SD4, *L. plantarum* CB2, and *L. plantarum* FCW4 isolates were Gram-positive, non-motile, rod-shaped bacteria with flat, circular, creamy-white colonies (Figure 2; Table 1). They were non-spore-forming and catalase-negative (Table 1).

### 2.2. Characterization of Fusarium Antagonism

**Figure 3:**
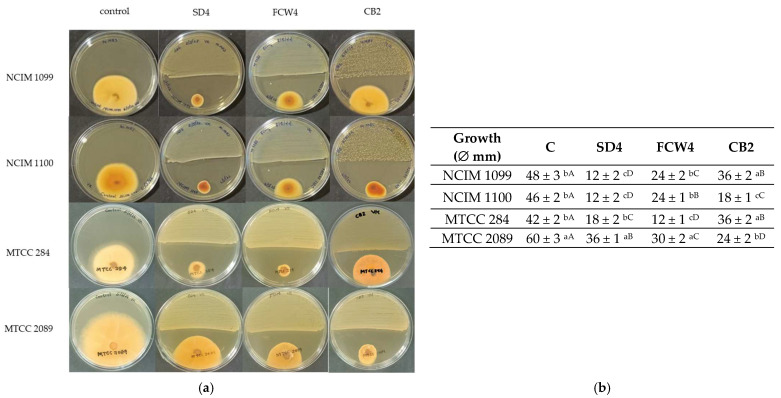
Antagonism test. Growth inhibition of different Fusaria by LAB strains. (**a**) *Fusarium verticillioides* NCIM 1099 and NCIM 1100, *F. oxysporum* MTCC 284, and *F. graminearum* MTCC 2089 strains were tested against *Limosilactobacillus fermentum* SD4, *Lactiplantibacillus plantarum* FCW4 and *L. plantarum* CB2 strains. (**b**) The mean inhibition-zone diameter and standard deviation are shown (n = 3). Significant differences are shown with different letters (*p* < 0.05). Lowercase letters compare the fungal growth results gained under the same LAB treatment. In contrast, uppercase letters show significant differences between the fungal cultures and LAB strain treatments.

To investigate the antagonism, the characterized LAB strains were co-cultured with Fusaria on the plate surface (Figure 3a). The LAB strains were found to inhibit the Fusaria species’ growth. Statistical analysis highlighted significant differences (*p* < 0.05) in colony formation between *F. graminearum* MTCC 2089 and other fungal strains. Specifically, the SD4 strain demonstrated similar, strong inhibitory effects against *F. verticillioides* NCIM 1099 and NCIM 1100 (75% and 73.9% inhibitions, respectively) but was significantly less effective against *F. oxysporum* MTCC 284 (57% inhibition) and *F. graminearum* MTCC 2089. FCW4 effectively inhibited *F. oxysporum* MTCC 284 (71.4%) and, while less potent against *F. verticilliodes* and *F. graminearum* strains, still achieved at least 50% inhibition against these species. CB2 demonstrated activity against both *F. graminearum* MTCC 2089 (60% inhibition) and *F. verticillioides* NCIM 1100 (60.8% inhibition) (Figure 3b).

**Figure 4:**
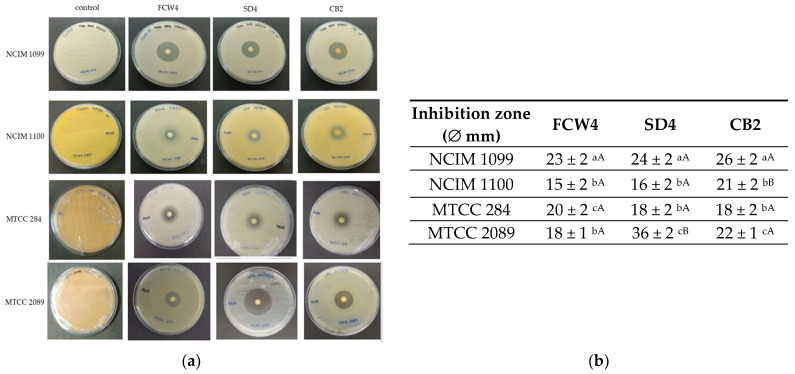
Disc diffusion test. (**a**) Antifungal activity of ethyl acetate extracts of different LAB strains on MHA plates, where 50 μL of crude ethyl acetate extract of bacterial culture fluid was loaded on sterile discs. *Fusarium verticillioides* NCIM 1099 and NCIM 1100, *F. oxysporum* MTCC 284, and *F. graminearum* MTCC 2089 strains were tested against *Limosilactobacillus fermentum* SD4 and *Lactiplantibacillus plantarum* FCW4 and CB2 strain extracts. (**b**) The mean inhibition-zone diameter and standard deviation are shown (n = 3). Significant differences are shown with different letters (*p* < 0.05). Lowercase letters compare the inhibition-zone results gained with the same LAB strain’s extract. In contrast, uppercase letters show significant differences between the inhibition zones gained on the same fungal strain.

Ethyl acetate extracts of the LAB cultures were tested for antifungal activity using a disc diffusion assay with equal extract volumes. Visual examination and halo measurements (Figure 4a) revealed that *F. verticillioides* NCIM 1099 was significantly more sensitive to concentrated LAB metabolites than NCIM 1100. All LAB strains exhibited strong inhibition (halos larger than 20 mm) against the NCIM 1099 strain. Furthermore, the SD4 extract significantly inhibited the growth of *F. graminearum* MTCC 2089 more effectively than extracts from the other strains (Figure 4b).

### 2.3. Resistance to Mycotoxins and Mycotoxin Reduction

Bacterial growth was investigated in liquid cultures to test the resistance of LABs against *Fusarium* mycotoxins (Table 2). Interestingly, DON inhibited only the FCW4 strain. For SD4, ZEA, T2, and FUM mycotoxins caused the same growth inhibition, significantly not different (*p* < 0.05) from each other. For the CB2 strain, ZEA caused a significantly higher growth inhibition than T2, FB1 or DON (Table 2). In the experiment, the SD4 strain was the most sensitive to all mycotoxins except DON. Mycotoxin reduction was also characterized in liquid LAB cultures. Analysis of the culture fluids revealed a significant (*p* < 0.05) decrease in ZEA concentration by *L. plantarum* FCW4 and CB2 under a one-hour treatment. For the other mycotoxins, the strains did not show significant mycotoxin reduction (Table 2).

### 2.4. Biocontrol Against Fusaria on Seeds and Crops

The antifungal effects of cell-free culture supernatants (CFCSs) from LAB strains were assessed against *Fusarium* contamination in various matrices, including disinfected and artificially contaminated peanuts, wheat grains, and corn kernels. Small-scale corn-silage fermentation was also conducted to evaluate the fermentation capabilities of LAB strains and their ability to reduce mycotoxin levels in mixed cultures.

#### 2.4.1. Antifungal Effect on Peanut

**Figure 5:**
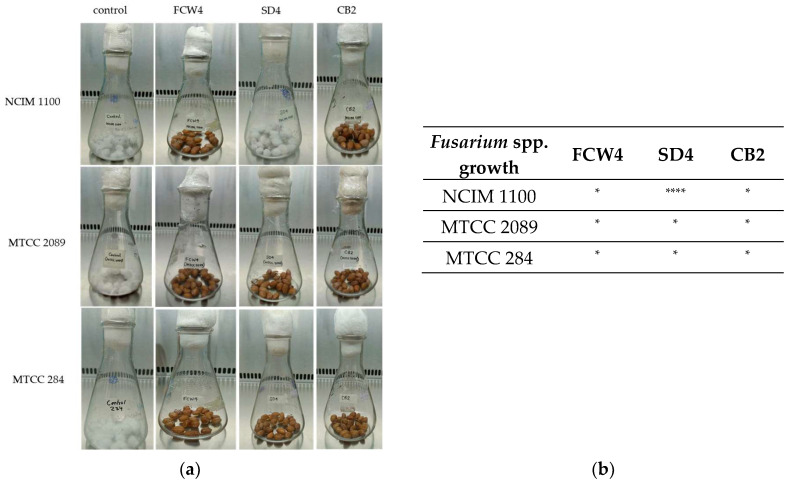
LAB antifungal effect was tested on surface-disinfected peanuts. (**a**) *Fusarium verticillioides* NCIM 1100, *F. oxysporum* MTCC 284, and *F. graminearum* MTCC 2089 strains were tested against cell-free culture supernatants (CFCSs) of *Limosilactobacillus fermentum* SD4 and *Lactiplantibacillus plantarum* FCW4 and CB2 strains’ culture fluids. Images showing the outgrowth of Fusaria on the surface-disinfected peanuts after 7 days. (**b**) Evaluation of *Fusarium* spp. growth with LAB CFCSs. *: no visual fungal contamination; ****: full contamination by Fusaria compared to controls.

The experiments with peanuts revealed a significant inhibitory effect of CFCSs. However, the CFCSs of the *L. fermentum* SD4 strain showed low antifungal activity against *F. verticillioides* NCIM 1100 (Figure 5), while the other treatments effectively inhibited the fungal growth on the peanuts after 7 days of incubation.

#### 2.4.2. Antifungal Effect on Wheat

**Figure 6:**
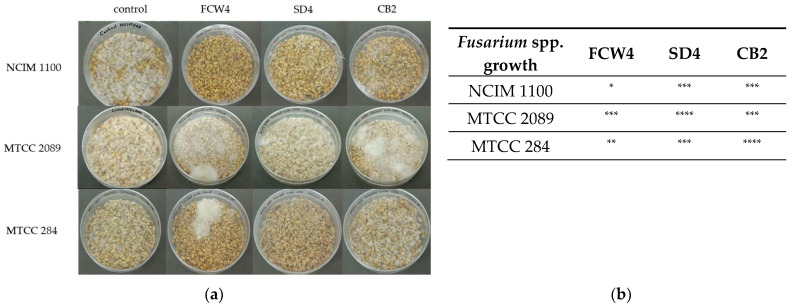
LAB antifungal effect tested on surface-disinfected wheat grains. *Fusarium verticillioides* NCIM 1100, *F. oxysporum* MTCC 284, and *F. graminearum* MTCC 2089 strains were tested against the CFCSs of *Limosilactobacillus fermentum* SD4 and *Lactiplantibacillus plantarum* FCW4 and CB2 strains. (**a**) The growth of contaminating Fusaria was inspected on 7-day-old cultures. (**b**) Evaluation of the fungal contamination. *: no visual fungal contamination; **: colony formation by Fusaria (or 25%); ***: medium contamination (or more than 50%); ****: full contamination by Fusaria compared to controls.

Surface-disinfected wheat grains were also treated with CFCSs of the LAB isolates and infected with the *Fusarium* spp. The resulting antifungal effect was variable. *F. verticillioides* NCIM 1100 and *F. oxysporum* MTCC 284 were the most sensitive to *L. plantarum* FCW4. Meanwhile, the other LAB strains showed medium or weak performance against the tested *Fusarium* strains (Figure 6).

#### 2.4.3. Corn Germination Test

A corn germination experiment was conducted to ensure the antagonism performance of LAB strains on corn kernels. First, the corn hybrid B73 was tested for endogenic fungal presence and found to be free from endogenic Fusaria. In the second step, we tested the plantlet’s development with and without a small cut close to the germ, which was applied to the kernels to help the fungal contamination. The treatments and the measurement results of the root and shoot lengths of the plantlets are presented in Table 3. Moreover, we tested LAB strains on cut kernels treated with *F. verticillioides* FGSC 7600 and compared the effects of the different CFCSs on plant development by measuring the root and shoot length. The results of the statistical analysis (Student’s t-test) are presented in Table 4.

The statistical analysis revealed that the cut close to the germ significantly increased the root and shoot length (treatments 1 and 2; Table 3 and Table 4). Moreover, the *F. verticillioides* FGSC 7600 treatment of the cut kernels caused a significant (*p* < 0.05) increase in shoot length (*p* = 0.0004) compared to cut kernels without fungal treatment (treatments 2 and 4). However, *F. verticillioides* FGSC 7600-treated cut and uncut kernels (treatments 3 and 4) did not show significant differences in plantlet development.

Any LAB treatments on *F. verticillioides* FGSC 7600-contaminated cut kernels (treatments 5, 6 and 7; Table 3) significantly increased the root lengths and significantly decreased the fungal mycelial growth compared to the development of LAB-free FGSC 7600 contaminated cut kernels (Table 4). These treatments resulted in different shoot lengths. With FCW4, the shoot length significantly decreased (*p* = 0.0108) compared to fungal-treated cut kernels, while with SD4 strain application, the shoot length increased (*p* = 0.0328). Moreover, treatment with the SD4 strain caused a significant increase in shoot length compared to the CB2 (*p* = 0.0030) and FCW4 (*p* = 0.0012) treatments. LAB treatments did not differ significantly in their effect, considering the fungal mycelial outgrowth on the kernels. With FCW4, CB2 and SD4 treatment, the fungal contamination (kernels fully or partially covered with mycelia) was 50 ± 1%, 60 ± 1% and 27 ± 2%, respectively.

#### 2.4.4. Silage Fermentation

The LAB isolates were also tested based on their performance in mixed cultures, as their antimicrobial activity can also affect beneficial bacteria in fermentations. The mycotoxin-treated and -untreated control silages differed significantly in their pH, LAB and mold counts, and acetic acid and lactic acid content (Table 5). Therefore, LAB treatments were only comparable to their corresponding controls.

Overall, the pH values were variable (pH 4.40–6.15). The lowest pH values were reached within the LAB treatments with SD4 and CB2 strains with mycotoxin supplementation. The highest lactic acid concentrations [22.6 ± 3.01 (m/m)% and 19.77 ± 2.96 (m/m)% for dry weight, respectively] without a significant difference were reached in the control silages (with and without mycotoxins). SD4 supplementation and mycotoxin treatment also ended with a lactic acid production (15.18 ± 2.26 ‘(m/m)%) that was statistically not different from its control. The SD4 strain and mycotoxin supplementation produced the highest LAB and lowest mold counts. The CB2 strain with mycotoxin supplementation resulted in statistically not different results for pH, LAB count, and acetic acid content compared to the SD4 treatment. However, with the application of CB2, after the silo box opening, the microbiological analysis revealed statistically high mold counts and also *Fusarium* spp. contamination. Meanwhile, *Fusarium* spp. was eliminated by the end of the fermentation with the SD4 strain. Mycotoxin addition inhibited the effect of FCW4 treatment on *Fusarium* outgrowth at the end of the fermentation, which can be explained by the long fermentation time or the inhibited LAB growth.

Neither DON, ZEA, nor FUM mycotoxins were detected in the non-supplemented samples at the end of the 6-week fermentation. Since in the supplemented raw plant material the total ZEA concentration (38.907 ± 4.2 µg/kg) was significantly lower (*p* = 0.03847) than the supplementation (141.5 ± 36.65 µg/kg ZEA), it was concluded that the raw plant material without prolonged incubation reduced the ZEA concentration (72.5% reduction). Considering the mycotoxin reduction in the mycotoxin-supplemented samples, significantly different ZEA concentrations were measured. With mycotoxin supplementations, the ZEA concentration was 23.59 ± 3.53 µg/kg in the final silage (83.3% reduction compared to the raw material). The FCW4 strain did not decrease the ZEA concentration to a lower level than the control sample after 6 weeks of ensiling. The SD4 strain was the best option for the fermentation at higher mycotoxin concentrations, and the silage resulted in a 90.8% ZEA decrease compared to the original and 54% difference compared to the control samples after 6 weeks of ensiling. The CB2 strain exhibited a greater decrease despite its weaker performance in the fermentation. The silage treatment resulted in a 94.1% ZEA decrease compared to the raw sample and a 64.7% difference from the control final silage. Interestingly, FUM concentrations did not differ significantly within the LAB treatments. The 2.13 mg/kg original FUM supplementation was radically reduced at the end of the 6 weeks. The total DON reduction was detected with the SD4 and CB2 supplementation. At the same time, FCW4 resulted in a reduced DON concentration that was not significantly different from the control silage. However, even in this case, the DON reduction was high (98%) compared to the original contamination.

## 3. Discussion

A variety of factors, including growth media, temperature and incubation time, pH, and nutritional factors, influence the antifungal substances and their production levels in LABs [14,15]. We applied stable conditions to compare the strains and select the best antagonist isolates. Following an in vitro screening of various LAB strains from diverse sources for antifungal activity, the best strains were selected for further studies. The *Lactiplantibacillus plantarum* FCW4 and CB2 strains and *Limosilactobacillus fermentum* SD4 were isolated, identified, and characterized to estimate their use as potential biocontrol agents. We conducted various experiments to assess the biocontrol potential, including plate assays and grain and fermentation tests. We also tested cell-free culture supernatants (CFCSs) to investigate the possibility of cell-free biocontrol.

Heterofermentative *Limosilactobacillus fermentum* isolates were found primarily in fermented foods/feeds and dairy products. These strains inhibited *Aspergillus* spp., *Penicillium* spp. [16,17,18], *Microsporum canis* [19], *F. graminearum* [20,21,22], *F. equiseti*, *F. proliferatum* [23] and *F. oxysporum* [24]. *L. fermentum* SD4 CFCSs performed well against *F. graminearum* MTCC 2089 in the disc test; however, in the plate co-culturing test, *F. verticillioides* was more sensitive to *L. fermentum* SD4 than *F. graminearum* MTCC 2089. *Fusarium* mycotoxins in 2 mg/kg concentration hindered SD4’s growth, and the mycotoxin reduction capability was low when the toxins were applied to a homogenous culture.

*Lactiplantibacillus plantarum* is a well-known probiotic LAB. It was isolated from various plant materials and dairy products and detected as an antimicrobial agent in the analyzed cultures [25,26]. *L. plantarum* cell-free extracts were effective against *Fusarium* species, including *F. avenaceum*, *F. culmorum*, *F. graminearum*, and *F. oxysporum* [27], similar to our results. *L. plantarum* strains inhibited *Fusarium* spore proliferation [28], and the cell-free extracts could also inhibit *Fusarium* metabolism [27]. The present experiments showed differences in antagonism among the LAB strains. Differences in the sensitivity of *F. verticillioides* strains, and the antagonist performance of *L. plantarum* FCW4 and CB2 were evident. We could conclude that even with cell-number normalization, different concentrations or cell metabolites produced in the CFCSs could cause variability in the antagonism.

Sathe et al. (2007) suggested that spraying highly diluted solutions of LABs onto plants and soil increases plant health and growth. For biocontrol, fresh vegetables were spray-inoculated with viable cell suspension or CFCSs of lactobacilli. The different spoilage fungi, like *F. graminearum*, were inhibited, and the fungal growth was significantly delayed [29]. However, the concept of plant protection with a biocontrol LAB application was developed much earlier [30]. A naturally fermented microbial mix was known to have a positive effect. Its microbiome was often dominated by photosynthetic bacteria, LABs, and yeasts [31].

In the wheat-treatment experiment, *L. plantarum* FCW4 showed a more potent antifungal effect than the CB2 or SD4 strains. The efficiency of LAB preparations was similarly inconsistent when comparing assays. Interestingly, Šaric et al. (2021), who also treated wheat with *L. fermentum*, mentioned that the media used in the experiment affected the activity or tolerance of the tested bacteria and fungi. However, the CFCSs of all the tested LAB species significantly inhibited the growth of *F. graminearum* (83–90% mean inhibition) but *Aspergillus niger* and *Penicillium chrysogenum* [32].

All LAB treatments reduced the *F. verticillioides* contamination of the corn kernels, and the selected LAB microbes could also increase the seedlings’ growing potential. Fungal growth inhibition was also observed in maize kernels over the control group after 7 days of incubation with a CFCS of *L. fermentum* MYSAGAM1. The inhibitory efficacy was considered due to the presence of organic acids [23].

*Lactiplatibacillus plantarum,* besides *Levilactobacillus brevis*, are well-known as inoculants for ensilaging; however, the aim of their usage usually lies in their high organic carbon production and not their biocontrol capability [33]. *L. fermentum* showed the potential to reduce mycotoxin contamination in sorghum silage effectively [34]. In the mixed culture of a corn silage [*L. fermentum* (61.2%), *L. plantarum* (11.2%), *L. paralimentaris* (11.2%), *L. pentosus* (5.6%), *L. buchneri* (5.6%), and *Sporolactobacillus* (5.6%)], and in the field as well, *L. fermentum* successfully inhibited *F. verticillioides* [35]. *L. fermentum* 5KJEU5 demonstrated an antifungal effect in kunu-zaki, a cereal-based indigenously fermented Nigerian beverage; therefore, it could prevent fungal growth and mycotoxin accumulation in cereal foods [18]. Considering LAB metabolism, a significant enhancement in antioxidant activity was reported in several LAB-fermented foods through phenol metabolism and individual acids [36]. 

The supplementation of *L. plantarum* FCW4 or *L. fermentum* SD4 in corn plant fermentation revealed low performance (low lactic acid, high mold count); however, this picture changed with mycotoxin supplementation. Interestingly, under mycotoxin supplementation together with the natural contaminations (free and masked mycotoxins) in mixed cultures, CB2 and FCW4 did not inhibit *Fusarium* outgrowth at the end of the fermentation, which can be explained by the long fermentation time or the inhibited LAB growth. *Limosilactobacillus fermentum* SD4 supplementation also resulted in different final silage quality with or without the mycotoxin addition. While SD4 supplementation alone was not the best option for the fermentation itself, with mycotoxin present, it was the most successful fermentation compared to the others. The pH, LABs, mold counts, acetic acid and lactic acid significantly differed from the control. Moreover, ZEA decreased under fermentation by 90.8% compared to the mycotoxin-supplemented raw corn plant.

Few studies have investigated mycotoxins in silages, and Driehuis, F. (2013) stated that evidence of fumonisin degradation in silage is lacking [37]. Nyamete et al. (2016) reported that *L. fermentum* reduced only 14% of Fumonisin B1 (a higher 20% reduction in the fermentation process) [38]. Chlebic et al. (2020) determined that fumonisin reduction by lactobacilli ranged between 62% and 77% [39]. Here, an average 85.7% decrease was detected with the LAB treatments, while, in a pure culture, it was under 15%. The highest reduction in concentration was observed for the ZEA (the highest reduction was 45.5% for CB2 in a pure culture, while in the SD4 mixed culture, it was 90.8%), while it was reported as 57% for lactobacilli [39]. By contrast, DON was the most resistant mycotoxin: its concentration was reduced by 19–39% by *Lactobacillus* spp. strains after 24 h of incubation [39]. DON is usually detected in silages, and its concentration can be very high (more than 2 mg/kg) [40]. Due to the biodegradation of DON conjugates (masked forms), a DON concentration increase could happen under corn ensiling [41,42]. Our experiments found DON under the detection level except with *L. plantarum* FCW4, where a low DON concentration was detected. The FCW4 and mycotoxin supplementation could inhibit some microbes, especially LABs with DON-degradation capabilities, as proved by the low LAB count and lactic acid in the FCW4 treatment. Moreover, DON also had potent inhibition (45.9%) on FCW4.

Applying LAB cultures in the field [31] or post-harvest [43] has proven to be efficient in reducing *Fusarium* contamination and increasing corn root length. In corn plant fermentation, the supplementation of *L. fermentum* SD4 with *L. plantarum* CB2 was suggested. For peanut and wheat treatment and mold control, *L. plantarum* FCW4 was identified as the best option. Developing cell-free products will help ensure the safe usage of the agents on cultivated products.

## 4. Materials and Methods

### 4.1. Materials

The potato dextrose media (PDA and PDB, Hi-Media, Mumbai, Maharashtra, India) were used to grow the *Fusarium* cultures. MRS agar and M17 media (Hi-Media, Mumbai, Maharashtra, India) were used to isolate and culture the lactobacilli. Mueller–Hinton Agar (MHA, Hi-Media, Mumbai, Maharashtra, India) medium was used for the antagonism studies to grow fungus and bacteria together. Similarly, a modified MRS medium without ammonium citrate and sodium acetate was also used to culture lactobacilli and *Fusarium* spp.

### 4.2. Microorganisms

Lactic acid bacteria (LABs) were isolated (34 isolates) from various sources such as sourdough, pickle, cabbage (fresh and old), cheese, goat dung, carrot, curd, bread, milk, fermented coconut water, etc., at NIIST, Trivandrum, Kerala, India. From the matrices, decimal dilution was prepared, streaked on MRS medium plates, and incubated at 37 °C for 24–48 h. Solitaire colonies were picked and streaked again onto MRS plates to prepare pure cultures. All growth inhibition assays that were performed at CSIR-NIIST, Thiruvananthapuram, India, were conducted with *Fusarium* strains used for seed infection assays as laboratory standards, namely *Fusarium verticillioides* NCIM 1099, *F. verticillioides* NCIM 1100, *Fusarium graminearum* MTCC 2089, and *Fusarium oxysporum* MTCC 284. *Fusarium verticillioides* FGSC 7600 was used in corn kernel germination tests. *Fusarium verticillioides* FGSC 7600 is a whole-genome-sequenced reference strain, and the assay was carried out at the accredited *Fusarium* laboratory of the University of Debrecen (Debrecen, Hungary), where all genetic, physiological, and virulence studies have been optimized with this strain. The fungal cultures grown in PDB were streaked to the PDA plates and incubated at 30 °C for 5–7 days.

### 4.3. Characterization of Lactic Acid Bacteria

The pure isolates were characterized by Gram staining and catalase tests. The Gram-positive and catalase-negative cultures were further tested for halo forming on modified MRS-calcium carbonate (0.8% *w/v* CaCO_3_) plates and incubated at 37 °C for 48 h [44]. Colonies positive for halo surroundings were selected and processed for a pure culture on MRS agar.

### 4.4. Molecular Identification

All bacterial strains were grown in MRS broth at 37 °C for 48 h under anaerobic conditions. The cultured cells were harvested by centrifugation at 13,600× *g* for 5 min, and the supernatants were removed. Genomic DNA was extracted using a bacterial genomic DNA extraction kit (G-Spin Intron Biotechnology, Seongnam, Republic of Korea) according to the manufacturer’s instructions. DNA concentration and purity were determined by the absorbance ratio A_260_/A_280_. The PCR amplification of the 16S rRNA bacterial barcode sequence was conducted using universal primers, and *Lactiplantibacillus plantarum* gene-specific primers (IDT, Leuven, Germany) were also applied [45]. We used the Phusion Hot Start II High–Fidelity DNA Polymerase (Thermo Fisher Scientific Life Technologies Inc., CA, USA), and the universal primers used were 1492R: 5′-GGT TAC CTT GTT ACG ACT T-3′, 515F: 5′-GTG CCA GCM GCC GCG GTA A-3′, Plantarum-F: 5′-GCT GGC AAT GCC ATC GTG CT-3′, Plantarum-R: 5′-TCT CAA CGG TTG CTG TAT CG-3′ [34]. Initial denaturation included 94 °C—10 min, 30 × 94 °C—30 s, 60 °C—30 s, 72 °C—30 s, and the final extension was 72 °C—5 min. The PCR products were checked in 1% agarose gel (1×TBE), then purified with NucleoSpin Gel and a PCR Clean-up kit (Macherey-Nagel, Düren, Germany) according to the manufacturer’s instructions. The resulting DNA sequences were determined (BIOMI Ltd., Gödöllő, Hungary). The data obtained were analyzed using the MEGA 11 (version 11.0.13.) program. The resulting sequences were submitted to the NCBI Gene Bank [46]. A homology search was performed with BLASTn: https://blast.ncbi.nlm.nih.gov/Blast.cgi (accessed on 11 March 2024). A phylogenetic tree was also built with the SD4 sequence. The tree was created at the website of Phylogeny.fr (http://www.phylogeny.fr, accessed on 22 January 2025), using Advanced mode with the default parameters (MUSCLE 3.8.31 for alignment, GBLOCKS 0.91b for curation of the alignment, PhyML 3.1 for phylogeny and TreeDyn 198.3 as tree viewer). The sequences of *Limosilactobacillus*-type strains and *Streptococcus pneumoniae* were downloaded from the NCBI database https://blast.ncbi.nlm.nih.gov/Blast.cgi (accessed on 22 January 2025).

### 4.5. Antagonism Tests

Different culture media like Muller–Hinton Agar (MHA), modified De Mann–Rogosa–Sharpe (MRS), M17, and Potato Dextrose Agar (PDA) media were used for the culturing of bacteria and fungi separately and also together in different combinations. LAB strains were cultured in MRS broth, while fungi were grown on PDA. Antagonism studies were conducted on either MHA or modified MRS medium.

A single bacterial colony selected from a fresh plate was suspended in the tube containing 250 μL sterile distilled water. Using a sterile cotton swab, the culture was spread over half of the surface of the modified MRS medium plates and incubated at 37 °C for 48 h. After incubation, the plates were taken, and using a sterile cork borer, a fungal disc was placed on the other half of the plates at one end and incubated at 30 °C for 5–6 days (n = 3). Control plates, which contained only fungal discs, were also prepared and incubated at 30 °C for 5–6 days. The plates were observed and compared with control plates to see any fungal growth inhibition by the bacteria.

### 4.6. Preparation of Bioactive Extracts and Disc Diffusion Assay

A 250 mL MRS broth was inoculated with a 24-hour-old bacterial culture (OD_600_ = 1) and incubated at 37 °C for 48 h in a static condition. After incubation, the supernatant was collected by centrifugation at 8590× *g* for 15 min. The supernatant was transferred to a 1L conical flask and a double volume of ethyl acetate and kept on a shaking incubator for one hour. After shaking, the extract was separated using a separating funnel, and the solvent phase was collected. The solvent was evaporated under reduced pressure in a rotary evaporator (BÜCHI, Flawill, Switzerland), and the semi-viscous concentrated sample was collected and adequately suspended. The obtained concentrated crude extract was used for disc diffusion assays. *Fusarium* spp. were grown in PDB and incubated for 2–3 days.

For the disc diffusion assay, the fungal suspension (0.1 mL) was uniformly spread on MHA plates, and 50 μL of crude extract was loaded on sterile discs and set on the fungal culture in triplicate. The inhibition zone was determined after incubation at 30 °C for 72 h.

### 4.7. Mycotoxin Detection by HPLC

HPLC measurements were performed on Thermo Scientific Dionex Ultimate 3000 (Dionex Softron Ltd., Germering, Germany) HPLC equipment. For all measurements, Biopure mycotoxin standard solutions (Romer Labs, Tulln, Austria) were used in an appropriate dilution [47]. To measure deoxynivalenol (DON), 12.5 g of dried sample was homogenized with 2.5 g polyethylene glycol (VWR International Ltd., Debrecen, Hungary) and 50 mL distilled water under high-speed stirring. After filtering, 1 mL of extract was poured onto the DON immunoaffinity column (VICAM DONtest HPLC column, Weber Consulting Ltd., Budapest, Hungary). The column was washed successively with 5 mL of distilled water and 5 mL of methanol to elute the toxin from the column. The methanol was evaporated from the samples on a rotary evaporator (BÜCHI, Flawill, Switzerland). During the HPLC test, a Phenomenex RP C18 column (125 × 4 mm, 5 μm) was used with a DAD detector at 218 nm with acetonitrile/water (10:90) eluent. For the HPLC measurement of zearalenone (ZEA), 20 g of dried sample was homogenized with 2 g sodium chloride (VWR, Debrecen, Hungary) and 50 mL 90% acetonitrile (HPLC grade, Merck GmbH, Darmstadt, Germany), stirring at high speed. Then, the extract was diluted with distilled water in a ratio of 1:4. The diluted extract was filtered, and 10 mL was filled into the zearalenone immunoaffinity column (VICAM ZearalaTest WB HPLC column, Weber Consulting Ltd., Göd, Hungary). The column was washed with 10 mL of distilled water, and the toxin was eluted with 5 mL of methanol. For the detection, a Phenomenex RP C18 column (150 × 4.5 mm, 5 μm) was used with ex360 nm, an em440 nm fluorescence detector, and an acetonitrile/water/methanol (46:46:8) elution. Thermo Scientific Dionex Chromeleon 7.2 Chromatography Data System (CDS) software (Dionex Softron Ltd., Germering, Germany) was used for data collection and evaluation. The performance, detection limit, linear range, and reproducibility of the applied HPLC methods were determined by the contamination of dried and ground maize silage with mycotoxins of different concentrations (n = 8). The LOD was 0.01 mg/kg DON and 0.001 µg/kg ZEA. A linear range of up to 50 mg/kg was detected. The relative standard deviation was calculated as the absolute value of the coefficient of variation, and in all cases, it was found to be below 10%.

### 4.8. Fumonisin Detection in ELISA Assay

The detection of FUM was performed using AgraQuant^®^ Fumonisin ELISA (Romer Labs, Tulln, Austria) according to the manufacturer’s instructions. Subsequently, using the BioTek Synergy HTX Multimode Reader (BioTek, Winooski, VT, USA), the samples were measured at 450 nm (n = 3, CV% < 5%; LOD 0.2 mg/kg).

### 4.9. Microbiological Analysis

During microbiological sample preparation, 100 g of sample was suspended in sterile homogenizing Stomacher bags in a ratio of 1:9 in buffered peptone water (BPW, Scharlab, Barcelona, Spain) and homogenized with a Stomacher homogenizer (IUL Instruments, Barcelona, Spain). Subsequently, further decimal dilutions were made from the suspensions. The lactic acid bacteria count was determined on De Mann–Rogosa–Sharp (MRS) Agar (Scharlab Hungary Ltd., Debrecen, Hungary) plates by surface dispersion method, and the plates were incubated at 37 °C under anaerobic conditions for 3 days. Mold counts were determined by surface dispersion on chloramphenicol-glucose agar (CGA) (Scharlab Hungary Ltd., Debrecen, Hungary) ) medium, which was incubated at 25 °C for 5 days.

### 4.10. Mycotoxin Resistance

BIOPURE mycotoxin solutions (Romer Labs, Tulln, Austria) were used in an appropriate dilution for all resistance tests. The cell cultures were inoculated in MRS medium (Scharlab) and incubated for 16 h at 37 °C to obtain exponential phase cultures. A microtiter plate made with 200 μL MRS medium was inoculated to obtain a low-density culture (OD_630nm_ within 0.1–0.2). Mycotoxins were added to the cultures in a 2 mg/L final concentration. The bacteria were incubated with mycotoxins for 24 h at 37 °C in a microtiter plate reader (Synergy HTX Multimode Reader, BioTek, Winooski, VT, USA). The optical density was read hourly at 630 nm after intense shaking (30 s). The growth curve of untreated cultures (without mycotoxins) was compared to mycotoxin-treated cultures (n = 4). Data were analyzed in Gen5 version 3.05 software (BioTek, Winooski, VT, USA) and Microsoft Excel version 2501 [47]. Based on the analyses, 5% growth inhibition was taken as the minimal level statistically different from the control (*p* < 0.05).

### 4.11. Mycotoxin Reduction Tests

Mycotoxins (BIOPURE, Romer Labs, Tulln, Austria) were used for mycotoxin reduction tests in appropriate dilutions. The mycotoxins were diluted with MRS medium. A 16 h MRS culture (37 °C) was performed. All tests were performed in three parallels with positive (without cells) and negative (without mycotoxins, solvent) controls. All mycotoxin-supplemented samples were incubated in MRS for 1 h at 25 °C by shaking (250 r.p.m.), then centrifuged (13,600× *g* for 5 min, 4 °C), and supernatants were removed and analyzed with HPLC. The supernatants were treated with methanol in a 1:1 ratio and vortexed at high speed. The HPLC detection of the tested mycotoxins was performed with Thermo Scientific Dionex Ultimate 3000 (Dionex Softron Ltd., Germering, Germany) equipment [47]. Based on the analyses, a 15% mycotoxin concentration decrease was taken as the minimal level statistically different from the control (*p* < 0.05).

### 4.12. Biocontrol Efficiency of the LAB Strains

#### 4.12.1. Peanut

In this study, 10 g of surface-sterilized (4% sodium hypochlorite, 1 min) peanuts were transferred to sterile conical flasks. LAB strains were inoculated into MRS broth (50 mL) at 37 °C for 16 h. OD-normalized (OD_600_ = 1) cultures were harvested by centrifugation at 13,600× *g* for 5 min (4 °C), and the supernatants were removed. Then, 2 mL of the prepared CFCS (10× concentrated) was added into each flask and mixed well. Controls were prepared by inoculating peanuts with 2 mL of sterile MRS broth. Fungal cultures on PDA were incubated at 30 °C for 7 days, and inoculums were made with 10 mL of sterilized water per PDA culture plate. After incubation at room temperature for 3 h, fungal inoculums of 200 µL (10^5^ spores/mL) of *Fusarium verticillioides* NCIM 1100, *F. oxysporum* MTCC 284, and *F. graminearum* MTCC 2089 strains were used for the 10 g surface-sterilized peanuts. The flasks were incubated at 30 °C for 7 days [48]. The growth of the Fusaria was detected and evaluated visually compared to the control cultures.

#### 4.12.2. Wheat

Wheat grains (40 g) were washed with distilled water, surface-disinfected with 1 (*w*/*v*)% sodium hypochlorite and again with distilled water, and air-dried. LAB strains were inoculated into MRS broth (50 mL) at 37 °C for 16 h. OD-normalized (OD_600_ = 1) cultures were harvested by centrifugation at 13,600× *g* for 5 min (4 °C), and the supernatants were removed. Then, 5 mL of the prepared cell-free supernatants (10× concentrated) was added to each Petri plate and mixed well. Controls were prepared by inoculating substrate with 5 mL of sterile MRS broth without a bacterial strain. Fungal cultures on PDA were incubated at 30 °C for 7 days, and inoculums were made with 10 mL of sterilized water per PDA culture plate. After incubation at room temperature for 3 h, 1 mL of a fungal inoculum (10^5^ spores/mL) of *Fusarium verticillioides* NCIM 1100, *F. oxysporum* MTCC 284, and *F. graminearum* MTCC 2089 strains were used for the 40 g surface-sterilized wheat grains. The flasks were incubated at 30 °C for 7 days. The growth of the Fusaria was detected and evaluated visually compared to the control cultures [49].

#### 4.12.3. Corn Germination Test

A hybrid corn (B73; Martonvásár, Hungary) was used in this experiment. The corn kernels were surface-disinfected in 70% ethanol for 5 min, followed by a short wash in sterile distilled water and then in 100% sodium hypochlorite solution for 15 min. After disinfection, the kernels were washed three times for ten minutes each in sterile distilled water to eliminate the disinfecting agent. The kernels were first tested for endogen *Fusarium* contamination on PDA plates. When it was proved that they were endogen *Fusarium*-free, in a new experiment, the disinfected kernels were cut to a depth of 0.5 mm close to the germ to facilitate fungal infection and soaked into *F. verticillioides* FGSC 7600 cell suspensions (10^3^ spores/mL) for 24 h. After treatment, the kernels were put onto the surface of one sterile Petri dish to eliminate excess microbe suspension. The *Fusarium*-contaminated kernels were soaked into 10^8^ CFU/mL LAB CFCS. After treatment, the kernels were put onto the surface of one sterile Petri dish to eliminate excess microbe suspension. Then, the kernels were put into sterilized glass jars of 330 mL volume that contained wetted paper towels. Each treatment was replicated six times with five kernels per replicate (n = 30). The glass jars were covered with caps and kept in culture room conditions at 22 °C and 800 lux light intensity, with a 16:8 photoperiod [50]. The experiment was evaluated after 5 days, and the length of the longest root, number of roots, and length of shoots of the developing plantlets were recorded. Fungal contamination was evaluated on kernels as 0—no outgrowth and 1—mycelia-covered kernels.

### 4.13. Forage Fermentation

The maize plant material was grown in the Faculty of Agricultural and Food Sciences and Environmental Management Demonstration Garden, University of Debrecen (Debrecen, Hungary). Its cultivation was assisted by the Precision Crop Production Research and Development Service Center, University of Debrecen (Debrecen, Hungary). Whole corn plants were collected during the milk phase of growth. The collected wet, chopped corn plant material was treated with 0.1% formic acid and mixed with reference samples containing *Fusarium* mycotoxins (Multitoxin in corn 10003627, BIOPURE, Romer Labs, Tulln, Austria). The matrix was artificially contaminated with 2.13 ± 0.715 mg/kg fumonisins (FUM), 0.625 ± 0.140 mg/kg deoxynivalenol (DON), and 141.5 ± 36.65 µg/kg zearalenone (ZEA) calculated for dry weight. Moreover, 16-hour-old LAB cell cultures were used for inoculation at a 5.5 × 10^8^ CFU/kg concentration. Formulations of silage were prepared: control, mycotoxin-contaminated, mycotoxin-contaminated-LAB-inoculated, and LAB-inoculated. The corn plants of different compositions were kept in airtight plastic boxes (~350 g raw weight/box) at room temperature for 6 weeks, under continuous pressure. At the end of fermentation, dry matter, microbiological quality, pH, volatile acid content, and mycotoxins were determined.

Fermented matrices were dried (60 °C ± 1 °C) in an oven (UN55, Memmert GmbH, Schwabach, Germany), and dry mass was calculated (n = 8; CV < 10%). Microbiological characterization was conducted on MRS plates for LABs and chloramphenicol-glucose agar (CGA, Scharlab Hungary Ltd., Debrecen, Hungary) plates for mold count determinations.

The lactic acid content of the fermented matrix was determined by an HPLC-DAD method, where 50 g of silage sample was weighed into a conical flask, sealed with a parafilm after adding 300 mL of water and stored at 4 °C overnight. It was filtered with pleated filter paper and a 0.45 μm pore Spartan syringe filter (Whatman GmbH, Dassel, Germany). The filtered sample was loaded to a Phenomenex (Torrance, CA, USA) RP-C18 column (125 × 4 mm, 5 μm) with a DAD detector at UV 218 nm and an acetonitrile/water (10:90) eluent. The LOD was 0.1 mg/kg.

For acetic acid, 50 g of fermented matrix was sealed with 300 mL of 1% phosphoric acid solution and stored overnight at 4 °C. Conditions for gas chromatography: Varian CP 3800 (Varian Inc., Walnut Creek, CA, USA) with Variant CP-Wax 52 CB, 30 m × 0.25 mm ID; 0.25 μm Colonna, and flame ionization detector (FID) at 230 °C. The carrier gas was 2.0 mL/min helium, with constant flow. The injector was set to 240 °C, split: 1:10, and the injected volume was 1 μL. The temperature program set was 95 °C, 14.5 °C/min, and 185 °C (holding: 2.5 min). The LOD was 0.002 (m/m)% (n = 3).

### 4.14. Statistical Analysis

Growth data analysis was performed in Gen5 3.05 software (BioTec) and Microsoft Excel version 2501 Analysis ToolPac Add-in, where a Tukey test (*p* ≤ 0.05) was performed for significance analysis.

## Figures and Tables

**Table 1:** Identification and characterization of the lactic acid bacteria isolates.

LAB Strains		Source	Gram Staining	Catalase Assay	MRS+ CaCO_3_	Accession Number *
FCW4	*Lactiplantibacillus* *plantarum*	Fermented coconut water	+	-	+	MT180563
CB2	*Lactiplantibacillus* *plantarum*	Cabbage	+	-	+	PP784682
SD4	*Limosilactobacillus* *fermentum*	Sourdough	+	-	+	PP535088

* Accession numbers for NCBI databank for 16S ribosomal RNA gene partial sequences.

**Table 2:** Growth inhibition of LABs by different *Fusarium* mycotoxins and mycotoxin reduction of the same mycotoxins were tested. DON: deoxynivalenol, ZEA: zearalenone, FB1: fumonisin B1 and T2 mycotoxins were applied in 2 mg/L to the selected LAB cultures: *Limosilactobacillus fermentum* SD4 and *Lactiplantibacillus plantarum* FCW4 and CB2 strains in MRS medium. The mean values and standard deviation are shown (n = 3). Significant differences are shown with different letters (*p* < 0.05). Lowercase letters are meant to compare the results gained from the LAB strains with the same mycotoxin treatment. In contrast, uppercase letters show the significant differences between the mycotoxin treatments of the same bacterial strain.

**Growth Inhibition (%)**	**DON**	**ZEA**	**T2**	**FB1**
FCW4	45.9 ± 2.29 ^aA^	37 ± 1.5 ^bB^	27.6 ± 1.7 ^bC^	28 ± 1.7 ^bC^
SD4	<5 ^bA^	47.1 ± 3.4 ^aB^	44.6 ± 2.9 ^aB^	44.9 ± 2.6 ^aB^
CB2	<5 ^bA^	33.8 ± 2.8 ^bB^	28.4 ± 1.9 ^bC^	25.3 ± 1.8 ^bC^
**Mycotoxin Reduction (%)**	**DON**	**ZEA**	**T2**	**FB1**
FCW4	<15 ^a^	36.7 ± 2.1 ^b^	<15 ^a^	<15 ^a^
SD4	<15 ^a^	<15 ^a^	<15 ^a^	<15 ^a^
CB2	<15 ^a^	45.5 ± 3.2 ^c^	<15 ^a^	<15 ^a^

**Table 3:** Corn plantlet’s root and shoot length analysis after different treatments with *F. verticillioides* FGSC 7600 (Fv) and LABs: *Limosilactobacillus fermentum* SD4 and *Lactiplantibacillus plantarum* FCW4 and CB2 strains.

Treatments	Kernel	Fungus	LAB	Root (mm)	Shoot (mm)
1	-	-	-	39.16 ± 9.40	7.53 ± 3.73
2	cut	-	-	51.47 ± 10.87	10.47 ± 2.65
3	-	Fv	-	49.25 ± 9.66	12.29 ± 7.54
4	cut	Fv	-	48.57 ± 14.35	15.13 ± 5.76
5	cut	Fv	FCW4	56.07 ± 10.52	11.43 ± 5.09
6	cut	Fv	CB2	57.27 ± 15.97	12.53 ± 5.64
7	cut	Fv	SD4	62.09 ± 15.19	20.45 ± 6.70

**Table 4:** Statistical analysis results of the corn plantlet’s root and shoot development. *t*-test for two samples, assuming unequal variances, was performed by comparing the different treatments (Table 3). *p* < 0.05 was considered as a statistically significant difference between the treatments.

		Root			Shoot		
Treatments		t Stat	t Crit	*p*	t Stat	t Crit	*p*
1	2	−3.7346	2.0301	0.0007	−2.8055	2.0369	0.0085
2	4	0.8035	2.0141	0.4259	−3.8358	2.0154	0.0004
3	4	0.2140	2.0076	0.8314	−1.6086	2.0086	0.1140
4	5	−2.3082	2.0057	0.0249	2.6370	2.0025	0.0108
4	6	−2.2190	2.0025	0.0305	1.7673	2.0017	0.0824
4	7	−2.5633	2.1098	0.0202	−2.3370	2.1199	0.0328
5	6	−0.3436	2.0086	0.7326	−0.7933	2.0025	0.4309
5	7	−1.2130	2.1448	0.2452	−4.0576	2.1448	0.0012
6	7	−0.8886	2.0930	0.3854	−3.4944	2.1199	0.0030

**Table 5:** Application of LABs in a corn plant fermentation. *Limosilactobacillus fermentum* SD4 and *Lactiplantibacillus plantarum* FCW4 and CB2 were inoculated separately onto a wet, chopped corn matrix. Eight silage formulations were prepared: control (C), LAB-inoculated (C+SD4, C+CB2 and C+FCW4). mycotoxin-contaminated (C+M), mycotoxin-contaminated and LAB-inoculated (C+M+SD4, C+M+CB2 and C+M+FCW4). The matrix was artificially contaminated with 2.13 ± 0.715 mg/kg fumonisins (FUM), 0.625 ± 0.140 mg/kg deoxynivalenol (DON), and 141.5 ± 36.65 µg/kg zearalenone (ZEA) calculated for dry weight. The silage samples (n = 8 per treatment) were analyzed after 6 weeks of fermentation. Significant differences (*p* < 0.05) are shown with different letters.

Treatments	pH	Log LAB *	Log Mold *	Mycoflora **	LA (m/m%)	AA (m/m%)	ZEA (µg/kg)	FUM (mg/kg)	DON (mg/kg)
C	5.74 ± 0.2 a	7.342 ± 6.342 a	6.362 ± 5.361 a	*Fusarium*	22.6 ± 3.01 a	4.12 ± 0.62 a	<0.001	<0.200	<0.010
C+SD4	6 ± 0.15 a	8.041 ± 7.380 b	6.415 ± 5.414 ab	-	1.98 ± 0.26 b	1.25 ± 0.19 b	<0.001	<0.200	<0.010
C+CB2	5.18 ± 0.12 bc	8.380 ± 4.681 c	5.415 ± 4.412 c	-	6.58 ± 0.98 c	1.68 ± 0.25 bc	<0.001	<0.200	<0.010
C+FCW4	5.27 ± 0.07 b	8.041 ± 7.041 b	6.477 ± 5.477 b	-	4.45 ± 0.67 d	3.09 ± 0.46 d	<0.001	<0.200	<0.010
C+M	4.40 ± 0.2 d	7.662 ± 6.662 d	6.653 ± 5.653 d	-	19.77 ± 2.96 ae	0.930 ± 0.14 e	23.59 ± 3.53 a	0.27 ± 0.040 a	0.012 ± 0.001 a
C+M+SD4	5.06 ± 0.14 c	8.519 ± 7.518 c	4.903 ± 3.903 e	-	15.18 ± 2.26 e	1.97 ± 0.29 c	12.9 ± 1.935 b	0.360 ± 0.054 a	<0.010
C+M+CB2	5.05 ± 0.15 c	8.431 ± 7.431 c	6.204 ± 5.204 f	*Fusarium*	8.94 ± 1.34 f	1.44 ± 0.21 bc	8.33 ± 1.249 c	0.350 ± 0.052 a	<0.010
C+M+FCW4	6.15 ± 0.11 e	7.672 ± 6.672 d	6.681 ± 5.681 d	*Fusarium*	2.98 ± 0.44 g	0.890 ± 0.13 e	20.2 ± 3.03 a	0.240 ± 0.036 a	0.013 ± 0.001 a

* log10 of counts (CFU/g). ** contamination after silo opening. LA: lactic acid; AA: acetic acid.

## Data Availability

The original genetic sequence data gained in the study are openly available in NCBI at the accession numbers MT180563, PP784682, and PP535088.
